# Notes from the Wards of Mercy Hospital

**Published:** 1870-05

**Authors:** C. W. Earle

**Affiliations:** House Physician


### The Clinic.

### NOTES FROM THE WARDS OF MERCY HOSPITAL.

By C. W. Earle, M.D., House Physician.

During the past three months, several cases of more than usual interest have been admitted into the hospital. The notes on the operations, and the treatment of these cases, may be interesting to those remote from hospital advantages.

Case — Bradley D. Saltor, aged 36, by occupation a tinner, admitted December 20th, 1869.

*History.*—About a month previous to admission, while engaged in building a dome at Carlinsville, Ill., he fell with the scaffolding. Was treated near his home till admission to the hospital.

Upon examination, a prominence was fouud in the dorsal region of the spine, total paralysis below this projection, urine and faces passing involuntarily, with occasional priapism.

He also had a very large and deep ulcer on the sacrum, and bed sores on his back and upper part of thighs. Appetite very poor.

*Diagnosis.*—Fracture of vertebræ.

*Treatment.*—An air bed was obtained for him as soon as possible, and, to improve the appetite, he was given tinct. cinchona and arom. sulph. acid. The large ulcer on sacrum and the bed sores were washed with tepid water, slightly carbolized, and dressed with cotton batting, saturated with oleum ricini and carbolic acid (10–1). Opiates were given at night as required.

*Present Condition, April 1st, 1870.* Paralysis continues, urine and faces are still passed involuntarily. Ulcer on sacrum entirely filled with new flesh and almost closed. All the bed sores that he had on admission are healed, and the integument is protected with adhesive plaster.

Notwithstanding our efforts to prevent any new bed sores, we have not been able to keep the heels from sloughing to some extent. They are prevented from coming in contact with any of the bedding, and various devices are resorted to, in order to keep them from getting worse. His bowels have been relieved from time to time of the retained matter, by injections, and his urine, which at times has been heavily loaded with the phosphate, causing very severe pain in the bladder, has been drawn with the catheter when necessary. Occasionally he has suffered great pain in the vicinity of the kidneys and the epigastrium, with very severe headache. When any periodicity in fever or chills have been noticed, he has had quinine, and the general internal treatment has been according to the indications. The patient has thus been kept along for nearly five months, without the supervention of death. Erichson, in his surgery, says, that these fractures, with displacement, are always fatal. Primarily and immediate from the injury, secondarily and remote, (1) as the result of changes in the body, dependent on continued loss of innervation; (2) from inflammatory action, spreading up the membranes, and in the cord itself, giving rise to effusion, etc.

From death by the injury, at the time it was received, he was spared; and the length of time that we shall be able to prolong his life, by alleviating his sufferings and combating complications, the future history of the case will decide.

CASE —. Mrs. M. N., aged 40, residence in city, admitted January 20th.

*History.*—Four days previous to admission, this woman fell down a flight of stairs, injuring herself severely. Her injuries were dressed by two surgeons of the city, who visited her regularly, while she remained at her home.

*Examination.*—When first seen, her elbow and forearm were enveloped in a large sling, but the hand protruding, it was noticed to be black and cold. She was partially delirious, and may have been somewhat under the influence of opiates, as she seemed to suffer very little pain. I immediately removed all the dressings, and, now, the very severe nature of the injuries were apparent.

*Diagnosis.*—Comminuted fracture of the lower third of both radius and ulna, with a very large wound involving the elbow-joint. The exact nature of this wound, the depressed condition of the patient rendered it impracticable to fully determine. It is not my province to criticise; but I wish to give the manner in which these injuries were dressed, and let each reader of the EXAMINER draw his own conclusion. The wound at the elbow was closed with sutures, and the end of a ligature displayed itself, showing that some artery had been ligated. The comminuted fracture was dressed by placing a roller bandage next the integument, which, in the swollen condition of the arm, cut off completely what little circulation of blood there may have been. Over this bandage, two splints, seven inches in length, were placed; and with another bandage, and the large sling mentioned before, the dressing was complete. The husband of the patient informs me that swelling commenced immediately, and that the pain was intense. The surgeons (?) were entreated to either take off the dressing or relieve the pain, in some way. These gentlemen informed the patient and her friends that everything was "all right;" and, in this condition, she was admitted to the hospital.

To attempt to save the arm was not now to be considered, and all our efforts were directed in rallying the powers of the patient, and supporting the system, in order to amputate where the line of demarkation should indicate. Prof. Andrews saw her often, and all the care and attention was given that was possible. Opiates were prescribed to quiet her pain, warm fomentation to help the circulation was made, a generous diet and stimulants were given — all the indications were met promptly, but to no avail.

*January 22.* Delirium traumaticum ensued, which was controlled by anæsthetics; stimulants continued.

*January 23.* Pulse growing weaker. Died at 3 o'clock P.M.

In January, three cases with aneurism were admitted, an unusual number to have at one time in our hospital.

One died from rupture of sac; the second, after an operation, and the third has been discharged, somewhat benefited. I subjoin the notes on the several cases.

CASE —. Pat. Quinn, aged 28, farmer, admitted January 24th.

*History.*—Last summer, while engaged in work that required lifting, he noticed a quick, sharp pain in his left side, followed by some soreness and pain. He was treated for rheumatism for some time; but, after some weeks, noticing a little pulsating, he came to Chicago for treatment, and was admitted to the hospital.

Immediately on being received, he was placed on the following treatment:—

℞. Iodidi Potass, ------------------------------ ʒiij.

Simp. Syrup, } āā, ------------------------ ℥ij.

Aqua, }

M. Sig. Teaspoonful every four hours. Opiates to relieve pain, and a diet that he relished.

A day or two following his admission, Prof. Davis delivered a very valuable clinic at his bedside, giving us the differential diagnosis between it and all diseases with which it might be confounded. This lecture was fully reported by Dr. S. A. McWilliams; and there remains for me to communicate, simply the notes on the case, till his death.

*January 28th.* At 10 o'clock to-day, the patient grew suddenly pale, was seized with very severe pain in abdomen, experienced great difficulty in breathing, extremities were cold, and no pulse could be detected at the wrist. It was supposed by the House Staff that the sac had burst, and that the patient would die immediately, yet nothing was left undone, and, under the use of the proper remedies, he slowly rallied.

During the next 48 hours, his pulse came up, and, with the use of opiates, he was kept quite comfortable. Prof. Davis now saw the patient, and expressed the opinion that the sac of the aneurism had ruptured, but the contents, instead of passing directly into the peritoneal cavity and causing death, had followed down behind that membrane; the artery being one of those so situated that this could take place.

*January 31st.* Discolored spots were seen along the left side, especially near the crest of the ilium—probably the blood from the aneurismal sac, infiltrating through the tissues. *10 o'clock P.M.* Is having great pain, growing pale, pulse very weak and fluttering. Died at midnight. No autopsy permitted.

CASE —. Dennis Quill, aged 50, laborer, admitted January 26th.

*History.*—Three months ago, while at work in the Stock Yards, near this city, he noticed a small swelling just below Poupart's ligament, on the right leg. Was treated with iodide potass; but the tumor gradually increasing, he is now admitted to hospital for an operation. Placed on tinct. iron for four days previous to operation, and until day of surgical clinic.

*January 31st.* Prof. Andrews operated, in presence of the students of the Chicago Medical College.

*Operation.*—A full anodyne having been given, the ether was administered. To insure complete and perfect anæsthesia, as the Prof. required this in such a difficult operation, a very little chloroform was given. An incision was made, about four inches in length, one inch above, and parallel with Poupart's ligament.

The integument and all the different fasciæ were taken up and skilfully divided, the epigastric artery carefully avoided, and the peritoneum disclosed. This membrane was gently pushed aside, and the artery could be seen in the bottom of the wound. A large vein could easily be seen passing obliquely over the artery, which was also avoided. A ligature was now placed around the artery, and the aneurismal tumor collapsed.

The wound was closed by suture and adhesive straps, and the patient put to bed. To assist in the establishment of the collateral circulation, he was placed on his left side, to avoid pressure of the vessels on the right. He was ordered tinct. iron, gtt. xx; quinine, grs. iv; four times a day; and as he was accustomed to the use of alcoholic drink, it was thought advisable to continue stimulants in small quantities. His pulse remained good, and he rallied well from the operation.

*Evening of 31st.* Gave morph. to ease pain and produce sleep. Pulse 94, temperature of leg 96° F.

*February 1st, morning.* Pulse 100, temperature of leg 96°. No pain or symptoms of any inflammation.

*Noon.* Pulse 112, respiration 28, temperature in axilla 103½°, temperature of leg 98°. Collateral circulation apparently well established. *Evening.* Pulse 108, temperature in axilla 101°, leg warm, no pain. Everything looked favorable.

*February 2d.* Patient died at 2.30 this morning. No autopsy permitted.

*Remarks.*—We can never know the exact cause of this man's death. He certainly did not die from the effects of the operation, or from peritonitis, for he rallied well from the first; and although at the last he had a little tympanitis, yet not at all dangerous, as his pulse and temperature both indicate. There are some indications, after his death, that pointed to internal hemorrhage. Large quantities of sanious matter and blood were discharged from the mouth and nostrils; and it is highly probable that the same condition of the arteries, which permitted the formation of the aneurism below Poupart's ligament, had given rise to another aneurism, hidden from view, which ruptured and caused his death.

CASE —. Pat. Nash, aged 35, laborer, admitted Jan. 19th.

*History.*—The patient that now claims our consideration had always been very healthy, till about three years ago, when he had an attack of rheumatism, the acute form lasting him some eight months. A short time after this, he had a kind of paralysis, but was cured by Dr. Davis. In the autumn of 1859, he felt better than for years previous, and went to Michigan, where he worked for one month digging a canal, and exposed to all the inclemency of the weather. He came home sick, and was treated, as he says, for rheumatic fever, liver complaint, and dyspepsia. Four weeks before he came to hospital, he noticed a pulsating tumor just above the umbilicus; and calling the attention of his medical attendant to the fact, was advised "*to buy a dollar's worth of leeches, and apply.*" The full notes on this case, from the time of his admission until his discharge, March 19th, would be tedious; and it will be sufficient for me to say, that for the first two weeks, he took iodide potass, grs. vi, every three hours, and, from that time, 20 grs., three times a day. He was supplied with anodynes, and given other remedies, *pro re nata.* During the course of treatment, his aneurism certainly did not enlarge, and it was thought by those entirely competent to judge, that it was somewhat reduced in size. It will at least encourage us in the use of iod. potass, in such cases.

This case was very important to us, as regards diagnosis. The chief symptoms of an abdominal aneurism are pain, either constant or paroxysmal, an impulse to the head, as it is placed over it, with or without a swelling, and a blowing sound or thrill, communicated by auscultation. There are several affections with which we are liable to confound an abdominal aneurism; but, by careful study, we may diagnose the malady quite certainly. In rheumatism, neuralgia, colic, and disease of the spine, we have pain referred to this region, but there is an entire absence of physical signs. A simple pulsation may also take place in the abdominal region, not due to aneurism, as in hysteria, pregnancy, or from heart disease. The history of the case, in hysteria, with absence of tumor and peculiar thrill, would be sufficient to exclude it, while the signs of pregnancy, both presumptive and positive, would show us that condition. A pulsation from disease of the heart would be detected by examination of the thorax, with the stethoscope, and the usual signs of organic mischief. A tumor, the result of an enlargement of any of the internal viscera, as the left lobe of the liver, cancer of stomach, disease of pancreas, etc., etc., may encroach on the aorta and cause a pulsation. The history of the case, percussion, and a close analysis of all the symptoms will, as a general thing, give us the correct diagnosis.

### SERVICE OF PROF. N. S. DAVIS.

Reported by S. A. McWILLIAMS, M.D.

*February 10th, 1870.*—CASE I.—A boy, aged 10 years, laboring under an attack of rubeola. As the case was at the stage of full development of the cutaneous eruption, the class were required to examine it minutely, with an explanation of the differences between it and the eruption of scarlatina, on the one hand, and small-pox, on the other. It was remarked, that the disease was of a specific character, having its definite period of premonitory fever, of eruption, and convalescence; and when uncomplicated by other diseases, seldom required the active interference of medicine. The patient should neither be stimulated with hot drinks, to force out the eruption, nor catharticised or depleted in any way, under the vain hope of relieving the fever. The first only adds to the discomfort of the patient and increases the danger of bronchial and pneumonic complications; and the latter retards the appearance of the eruption, unnecessarily debilitates the patient, and often induces diarrhœa or dysentery, in the latter stages of the disease.

Patients laboring under rubeola should be kept quiet, in a comfortable temperature, reasonably well ventilated, and allowed simple, light nourishment and cooling drinks.

If the cough is very harsh and there is much soreness in the chest, an anodyne and expectorant mixture, consisting of—

℞. Comp. Syrup, Squills, -------------------- ℥iss.

Camph. Tinct. Opii, ---------------------- ℥iss.

mixed, may be given, in doses of one teaspoonful to adults, every three or four hours. The severer class of cases will also be benefited by a powder of six or eight grains Dover's powder, and one of calomel, each night, during the first three days, and and followed by a laxative, as soon as the eruption is fairly established on the skin. If, in the progress of the disease, it becomes complicated with either broncho-pneumonia, diarrhœa, or dysentery, these affections must be treated with their appropriate remedies.

NEPHRITIS, OR ACUTE BRIGHT'S DISEASE.

CASE II.—G. H., aged 22, was admitted into the hospital after the acute symptoms had passed. His body is universally dropsical; the whole surface blanched and tumefied. The countenance is pale and puffy, with a heavy, stupid expression. While he sits up, his feet and ankles are more swollen, if he lies down, his face and eyelids swell. His water is nearly one-third albumen, scanty, and of high specific gravity. Dropsy is not a disease, but only a symptom, in this case, of inflammation of the kidneys. As the albumen escapes, it leaves the blood less viscid and more watery. The blood corpuscles diminish in number as the disease proceeds. Exosmosis now readily takes place, and the watery element obeys the laws of gravitation, and seeks the most dependent portions. Much benefit is frequently derived, in this stage, from a dr., four times daily, of the following:—

℞. Fl. Ex. Scutellaria,----------------------- ℥iij.

Acetate Potass, -------------------------- ʒiv.

Tinct. Digitalis,---------------------------- ℥j.

If stomach is irritable and appetite poor, benefit may be derived from—

℞. Subnitrate Bismuth, ------------------ gr. v.
Lupuline,-------------------------------- gr. iss., every six hours.

In the latter stages, the ferruginous preparations are found to improve the tone of the capillaries. When the kidneys do not secrete urine, for want of sufficient activity of the vaso-motor nervous system, and the bowels are bloated with gas, from the same cause, small doses of nitric acid and strychnine frequently produce very beneficial effects, both by increasing the urinary secretion and the action of the muscular coat of the bowels. The warm bath, two or three times per week, is also a material aid in determining to the skin and relieving the kidneys.

TYPHOID DYSENTERY.

*February 17th.* Prof. N. S. Davis called the attention of the class to a middle-aged lady, admitted to the hospital recently. She had complained of great pain in the right side, accompanied by nausea. The pain was in that locality where the liver, gall-bladder, and colon touch each other, so that it might be referred to either, or to rheumatism of the muscles covering them. There was no enlargement of the above structure, as there was no evidence that the patient ever had rheumatism, in any part of the body. To subdue what was supposed to be a local inflammation, the following was prescribed every four hours:—

℞. Sulph. Morph, ---------------------------- gr. $\frac{1}{3}$

Hydrg. Chlor. Mitis, -------------------- gr. ij.

After four powders, the bowels were found to be frequently opened. The discharges were bloody serum, with very little fœcal matter. The abdominal pains were severe, the tongue coated and brown, the skin congested, indicating feeble capillary circulation, and the stomach was still irritable. She was directed a powder of tannate of quinine, two grains, and sulphate of morph., one-quarter of a grain, every four hours, and a teaspoonful of an emulsion of oil of turpentine and tincture of opium between. Beef-tea and milk porridge for nourishment. During the succeeding 24 hours, the pain abated, but the intestinal discharges continued frequent, and of the same dark, bloody serum, with a cool skin, and feeble, quick pulse. The turpentine and laudanum emulsion was continued, with the addition of eight drops of chloroform to each dose; but instead of the powders of tannate of quinine and morphine, she took a teaspoonful of the following, just as often:—

| | | |
|---|---|---|
| ℞. | Strychnine, | 1 gr. |
| | Nitric Acid, | ʒj. |
| | Simple Syrup, | ℥j. |
| | Water, | ℥ij. |

Mix, and take in sweetened water.

She was also directed to have an enema of acetate of lead, 10 grains; tinct. opii, ʒss; and cold water, ℥ij, after every second evacuation of the bowels. Under this treatment, her symptoms began to improve. The intestinal discharges were less frequent and without blood; the pulse more full and less frequent; the extremities warmer, and the skin better color. She retained the emulsion, and chloroform, and nourishment, in small quantities, but complained much of the strychnine solution which was discontinued. At the present time, the skin generally is cool, but the capillary circulation good; pulse 100 per minute, and weak; abdomen not tympanitic; tongue and mouth moist, and bowels quiet. The symptoms of the patient were represented as indicating the commencement of convalescence, but accompanied by great feebleness, with decided impairment of the texture of the mucous membrane, of the lower half of the intestinal canal. The leading objects of treatment now should be to reëstablish digestion and nutrition, and to allay the sensitiveness of the mucous membrane, until it had more fully recovered the integrity of its texture. To accomplish these objects, the Lecturer advised the continuance of a porridge of sweet milk and wheat flour, in small quantities, for nourishment, and a powder of sub. nitrate of bismuth, 6 grs, and sulph. morph, $\frac{1}{6}$ gr., every 4 hours. And if there occurred any nausea, to allay it by teaspoonful doses of the following:—

| | | |
|---|---|---|
| ℞. | Aromat. Spirits Ammon, | ʒiij. |
| | Tinct. Opii *et* Camph., | ℥j. |
| | Simple Syrup, | ℥j. |
| | Aqua, | ℥j. |

Mix.

*P.S.*—Several weeks have elapsed since the above was written, and we learn that this patient continued to improve daily, until she was discharged well.

